# *In situ* template synthesis of hierarchical porous carbon used for high performance lithium–sulfur batteries†

**DOI:** 10.1039/c7ra12978e

**Published:** 2018-01-24

**Authors:** Lizhen Long, Xunyuan Jiang, Jun Liu, Dongmei Han, Min Xiao, Shuanjin Wang, Yuezhong Meng

**Affiliations:** College of Physics and Technology, Guangxi Normal University Guilin 541004 P. R. China longlzh@foxmail.com +86-20-84114113 +86-20-84114113; The Key Laboratory of Low-carbon Chemistry & Energy Conservation of Guangdong Province, State Key Laboratory of Optoelectronic Materials and Technologies, Sun Yat-sen University Guangzhou 510275 P. R. China mengyzh@mail.sysu.edu.cn handongm@mail.sysu.edu.cn

## Abstract

Hierarchical porous carbon (HPC) consists of micropores, mesopores and macrospores which are synthesized by *in situ* formation of template followed by acid etching. The obtained pores are three-dimensional and interconnected, and evenly distributed in the carbon matrix. By adjusting the ratio of the raw materials, the high specific surface area and large pore volume is afforded. The obtained HPC-3 samples possess graphite flakes and locally graphited-carbon walls, which provide good electrical conductivity. These unique characteristics make these materials suitable cathode scaffolds for Li–S batteries. After encapsulating 61% sulfur into HPC-3 host, the S/HPC-3 composite exhibits excellent cycling stability, high columbic efficiency, and superior rate cycling as a cathode material. The S/HPC-3 composite cathode displays an initial discharge capacity of 1059 mA h g^−1^, and a reversible capacity of 797 mA h g^−1^ after 200 cycles at 0.2C. The discharge capacities of the S/HPC-3 composite cathode after every 10 cycles at 0.1, 0.2, 0.5, 1, and 2C are 1119, 1056, 982, 921, and 829 mA h g^−1^, respectively.

## Abstract 2

*In situ* template synthesis of HPCs used for lithium–sulfur batteries, which exhibits excellent cycling stability and superior rate cycling.

## Graphical abstract

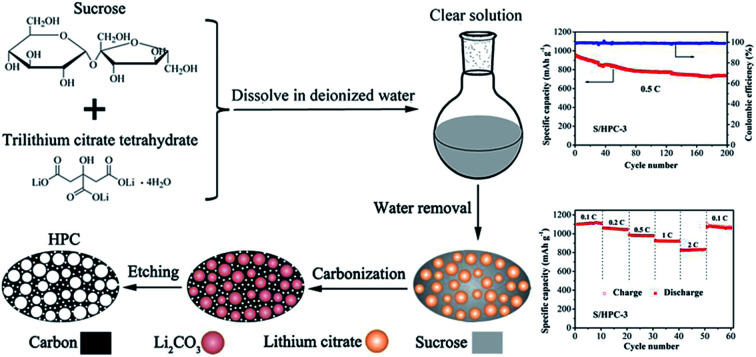

## 1. Introduction

Rechargeable batteries with high energy density, long-term durability, improved safety, and in particular low cost are expected to power future portable electronic devices, electric vehicles and large-scale smart grids.^1–3^ Lithium–sulfur (Li–S) batteries are considered as one of the most promising candidates for next-generation rechargeable batteries due to their high theoretical specific capacity (1672 mA h g^−1^) and high energy density (2567 W h kg^−1^), which are nearly five times higher than those of commercially available lithium-ion batteries.^4–7^ Additionally, sulfur element is earth-abundant and nontoxic, and as cathode active materials can largely reduce the overall production cost.^4,6–9^ However, some big challenges associated with the sulfur cathode need to be solved before its large-scale commercial application. Firstly, sulfur element is a nonconductor with an electric conductivity of 5 × 10^−30^ S cm^−1^ at 25 °C.^10^ The insulation characteristic of sulfur severely limits the sulfur utilization and influences the rate performance of lithium–sulfur batteries.^2,11^ Secondly, sulfur is reduced stepwise by lithium to a sequence of lithium polysulfide intermediates (Li_2_S_*n*_, 3 ≤ *n* ≤ 6) during the discharge process. These intermediates are highly soluble in the organic electrolyte, and can take part in the well described “internal shuttle process”, resulting in the loss of active material and low coulombic efficiency.^12,13^ Thirdly, the cathode suffers from significant volume variation during charge/discharge process as the result of density difference between S (1.96 g cm^−3^) and Li_2_S (1.66 g cm^−3^). These changes may deteriorate the electrode structure and result in capacity decay.^4,6,9^

In the past decades, numerous novel sulfur cathodes have been extensively explored to address the aforementioned issues, including carbon–sulfur composites (*e.g.*, porous carbon,^14–18^ carbon nanotubes,^19–21^ carbon nanofibers,^22,23^ graphene^24–26^ and graphene oxide,^27^*etc.*), conductive polymer–sulfur composites^28^ (*e.g.*, polypyrrole (PPy),^29,30^ polyacrylonitrile (PAN),^31,32^ polyaniline (PANI),^33,34^*etc.*), sulfur–metal oxide composites (*e.g.*, SiO_2_,^7^ TiO_2_,^35^*etc.*). Among these hosts, porous carbons have been proved to be effective and facile candidates due to its excellent electrical conductivity, large specific surface area, and abundant porous structure. The porous structure of the carbon matrix could effectively encapsulate sulfur and restrain the solubility of polysulfides. And their high specific surface area could provide efficient contact between sulfur and carbon for sufficient sulfur utilization. Based on their pore size, the porous carbons can be classified as microporous (*D* < 2 nm), mesoporous (2 nm < *D* < 50 nm), and macroporous (*D* > 50 nm) carbon. And each type of porous carbon possesses unique morphological advantages. The microporous and mesoporous carbon can increase the contact area between sulfur and carbon and provide active sites for electrochemical reaction. In addition, the microporous carbon is beneficial to trap polysulfide and lead to a stable cycling performance. But, such microporous carbon can only accommodate low sulfur content (less than 50 wt%), which limits the energy density of the Li–S battery. While, the macroporous carbon can raise sulfur loading, reduce diffusive resistance to mass transport, and accommodate the volume variation during cycling process. However, carbon hosts containing macropores or mesopores with large-size would suffer from the dissolution of polysulfide, which will lead to the large capacity loss during cycling. Hierarchical porous carbon comprised of macro-, meso-, and micro-pores may achieve good electrochemical performance if well combined with sulfur due to their synergistic effects. Nazar *et al.*^18^ encapsulated up to 70 wt% sulfur into highly ordered channels of CMK-3 to achieving intimate contact of carbon and sulfur. And a reversible capacities up to 1000 mA h g^−1^ at a current rate of 0.1C (1C = 1675 mA g^−1^) have been achieved. Xiaogang Zhang *et al.*^36^ prepare a three-dimensional (3D) hierarchically ordered porous carbon with mesoporous walls and interconnected macropores to encapsulate sulfur for high-performance Li–S batteries. The HOPC/S with well-defined nanostructure delivers a high initial specific capacity up to 1193 mA h g^−1^ and a stable capacity of 884 mA h g^−1^ after 50 cycles at 0.1C.

To date, the major challenge is to obtain hierarchically porous carbon with very high surface area, large pore volume and hierarchical porosity. Current synthetic methods can be categorized as hard-template, soft-template and non-template methods.^37^ The hard template method (nanocasting method) is the most commonly adopted method for preparing hierarchically porous carbon with well-defined pore structures and narrow pore-size distributions.^15,38–41^ It is generally accepted that, in this strategy, micropores and small mesopores are generated by releasing gases (CO_2_, H_2_O, CO, *etc.*) in the process of thermal decomposition of carbon sources, and large mesopores or macropores are replicated from hard templates.^15,41,42^ Zhang *et al.*^40^ have synthesized HPC by using Mg(OH)_2_ as template and soluble starch as carbon source. The as-prepared HPC exhibits a relatively high specific surface area of 902.5 m^2^ g^−1^ and large total pore volume of 2.60 cm^3^ g^−1^. When evaluated as cathodes for Li–S batteries, the S/HPC composite exhibited superior electrochemical performance. Wang *et al.*^39^ have prepared a series of large mesoporous carbons with hierarchical porosities using CaCO_3_ nanoparticles as template, formaldehyde resin as carbon precursor. By adjusting the radio of CaCO_3_ to formaldehyde resin, the highest BET surface area (1215 m^2^ g^−1^) and pore volume (9.0 cm^3^ g^−1^) have been obtained. Zhang *et al.*^15^ obtained a HPC material by using sucrose as the carbon source, CaCO_3_ as the template, and (CH_3_COO)_2_Cu·H_2_O (Cu(Ac)_2_) as the additive. The addition of Cu(Ac)_2_ influences the carbonization process, resulting in the volume increment of small mesopores. Thus, a better utilization of sulfur is achieved and the initial discharge capacity increases from 1287 to 1397 mA h g^−1^. Li *et al.*^41^ have prepared mesoporous carbon (MC) spheres with hierarchical pores, controlled pore volume and high specific surface areas using sodium alginate as carbon precursor and colloidal silica particles as template. After impregnating 60 wt% sulfur, the obtained S/MC composite cathode material displayed a high initial discharge capacity of 1388 mA h g^−1^ and a good cycling stability of 857 mA h g^−1^ after 100 cycles at 0.2C, and shows also excellent high rate capability.

**Fig. 1:**
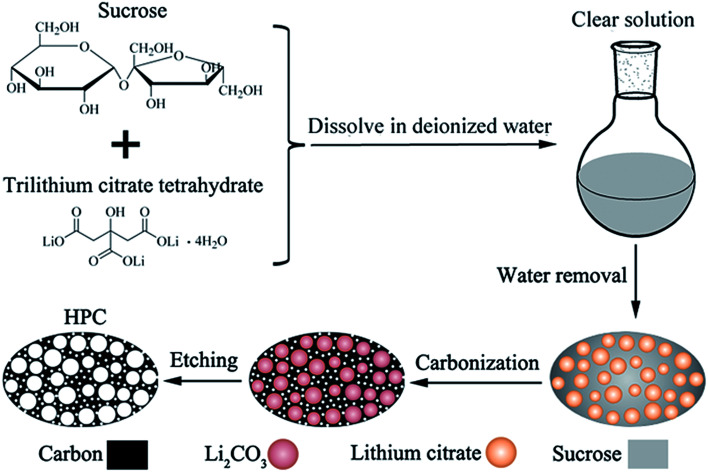
Schematic drawing for the synthesis of HPC by *in situ* formation of template in carbon matrix.

For preparing porous carbon materials using hard-template method, the aggregation of nanoparticles is the major problem due to their high surface energy, which results in the formation of large quantities of macropores. In this paper, this problem can be effectively avoided *via in situ* formation of template in carbon matrix, as displayed in the process scheme in Fig. 1. In this work, water-soluble lithium citrate is used as template precursor. Sucrose and lithium citrate are both acted as carbon sources. Firstly, the lithium citrate and sucrose are dissolved together in deionized water to form a homogeneous and clear solution. Subsequently, water was gradually evaporated to make the dissolved lithium citrate recrystallize from viscous sucrose solution and finally *in situ* locked within the sucrose matrix. In the step of carbonization, lithium citrate is decomposed into lithium salt with uniform dimension and high dispersibility. After etching lithium salts away by acid, the HPC comprised of micropores, mesopores and macropores are obtained. The HPC samples possess graphite flakes and graphited-carbon walls, which provide good electric conductivity. The unique characteristics of high electric conductivity, high specific surface and high pore volume endow the HPC suitable cathode scaffolds for Li–S batteries.

## 2. Experimental section

### 2.1 Chemicals and materials

All reagents were of analytical grade and used as received. The trilithium citrate tetrahydrate and sucrose were purchased from Sinopharm Chemical Reagent Co., Ltd. The sodium hydroxide (NaOH, ≥96%) and hydrochloric acid (HCl, 36–38%) were obtained from Guangzhou Chemical Reagent Factory.

### 2.2 Synthesis of hierarchically porous carbon (HPC)

HPC samples were prepared by *in situ* formation of template followed by acid etching strategy. Water-soluble lithium citrate is used as template precursor. Sucrose and lithium citrate are both acted as carbon sources. The preparation process is as follows: firstly, 1 g of sucrose and a certain amount of trilithium citrate tetrahydrate (sucrose : trilithium citrate tetrahydrate = 1 : 0.77, 1 : 1.54, 1 : 2.31, and 1 : 3.08, denoted as HPC-1, HPC-2, HPC-3 and HPC-4, respectively) were dissolved in 50 ml deionized water together with 0.1 ml of 2.5 M NaOH aqueous solution to form a homogeneous solution. Secondly, the deionized water was removed by vacuum-rotary evaporation method to obtain a sticky mixture. Thirdly, the mixture was placed in a 100 °C vacuum oven for 3 h and then 160 °C for 6 h to get a brown product. Subsequently, the brown product was carbonized in a tubular furnace under N_2_ flow at room temperature to 950 °C at 5 °C min^−1^. The salts obtained from the carbonization of trilithium citrate tetrahydrate were characterized to be Li_2_CO_3_, as confirmed by the XRD pattern of HPC-3 before acid-treatment, (see Fig. S1†). As shown in SEM images of Fig. S2,† the Li_2_CO_3_ salts (white dots) are dispersed in carbon matrix with uniform dimensions and high dispersibility. Finally, the Li_2_CO_3_ templates were etched away by 2 M hydrochloric acid to obtain the HPC samples. Thermal gravimetric analysis confirmed that Li_2_CO_3_ salts are completely etched away for all HPC samples, as shown in Fig. S3.†

### 2.3 Synthesis of S/HPC-3 composite

Due to the highest specific surface area and largest pore volume among the four samples, the HPC-3 is chosen as the host for encapsulating sulfur to prepare cathode materials. The S/HPC-3 composite was prepared following the typical melt-diffusion strategy. The HPC-3 and sulfur with a weight ratio of 1 : 2 were ground together and heated in a tube furnace at 155 °C for 12 h followed by another 2 h at 220 °C under N_2_ protection. After cooling down to room temperature, the S/HPC-3 composite was obtained.

### 2.4 Materials characterization

N_2_ adsorption/desorption analysis was performed on a Micromeritics ASAP 2460 analyzer at −196 °C using nitrogen. Electron conductivity was measured on a semiconductor powder electron conductivity test board (Suzhou Jingle Electronic Co., Ltd., SZT-D) at ambient temperature using the four-contact method. Thermogravimetric analysis (TGA) was conducted on PerkinElmer Pyris Diamond TG/DTA thermal analyzer. The X-ray diffraction (XRD) was operated at 40 kV and 40 mA by a D/Max-IIIA Powder X-ray diffractometer and using Cu-Kα radiation (*λ* = 0.15406 nm). Raman spectra were recorded using a Renishaw inVia Raman spectrophotometer with a He–Ne laser excitation at 633 nm. The morphology of the samples was examined by a field emission environment scanning electron microscope (SEM, Quanta 400F) and transmission electron microscopy (TEM, FEI Tecnai G2 F30) with an acceleration voltage of 300 kV. An energy dispersive spectrometers (EDS) attached to the TEM apparatus was used for microscopic elemental analysis.

### 2.5 Electrode preparation and electrochemical measurements

Cathode from the S/HPC-3 composite was prepared by a slurry coating procedure. The S/HPC-3 composite was mixed with super-P and polyvinylidene fluoride (PVDF) in a weight ratio of 90 (composite) : 2 (super P) : 8 (binder), and *N*-methylpyrrolidinone (NMP) was added to form homogeneous slurries. After ball-milling at a rotate speed of 160 rpm for 24 h, the slurries were cast onto the carbon-coated aluminum foil using the doctor blade method and dried at 60 °C overnight. Then, the electrodes were punched into 12 mm circular discs and the sulfur loading was calculated to be about 2.0 mg cm^−2^. For comparison, the pure sulfur electrode, was prepared by the same procedure but based on sulfur, super P, and PVDF in a mass ratio of 60 : 32 : 8. CR2025 coin-type half-cells were assembled in an argon-filled glove box (Mikrouna super 1220/750). 1 M lithium bis(trifluoromethane sulfonyl)imide (LiTFSI) in 1,3-dioxolane (DOL) and 1,2-dimethoxymethane (DME) (1 : 1 = v/v) containing 2 wt% LiNO_3_ was used as the electrolyte. The amount of the electrolyte in the cell is 50 μl. Lithium metal was used as the counter electrode and a microporous polyethylene membrane (Celgard 2500) was used as separator.

Galvanostatic charge–discharge tests were performed using a CT2001ALand Battery Testing System in the potential range of 1.7–2.8 V *versus* Li/Li^+^ at different current densities. All specific capacity values were calculated on the basis of sulfur mass. Cyclic voltammetry (CV) measurements were carried out using an S1287 electrochemical interface (Solartron) in the potential range of 1.7 to 2.7 V *versus* Li/Li^+^ with a scan rate of 0.2 mV s^−1^. Electrochemical impedance spectroscopy (EIS) was recorded on an electrochemical working station PCI4/300 (Gamry Instrument, Warminster, PA, USA) in the range of 1 MHz to 0.01 Hz with a disturbance amplitude of 5 mV. All electrochemical tests were conducted at room temperature.

## 3. Results and discussion

**Fig. 2:**
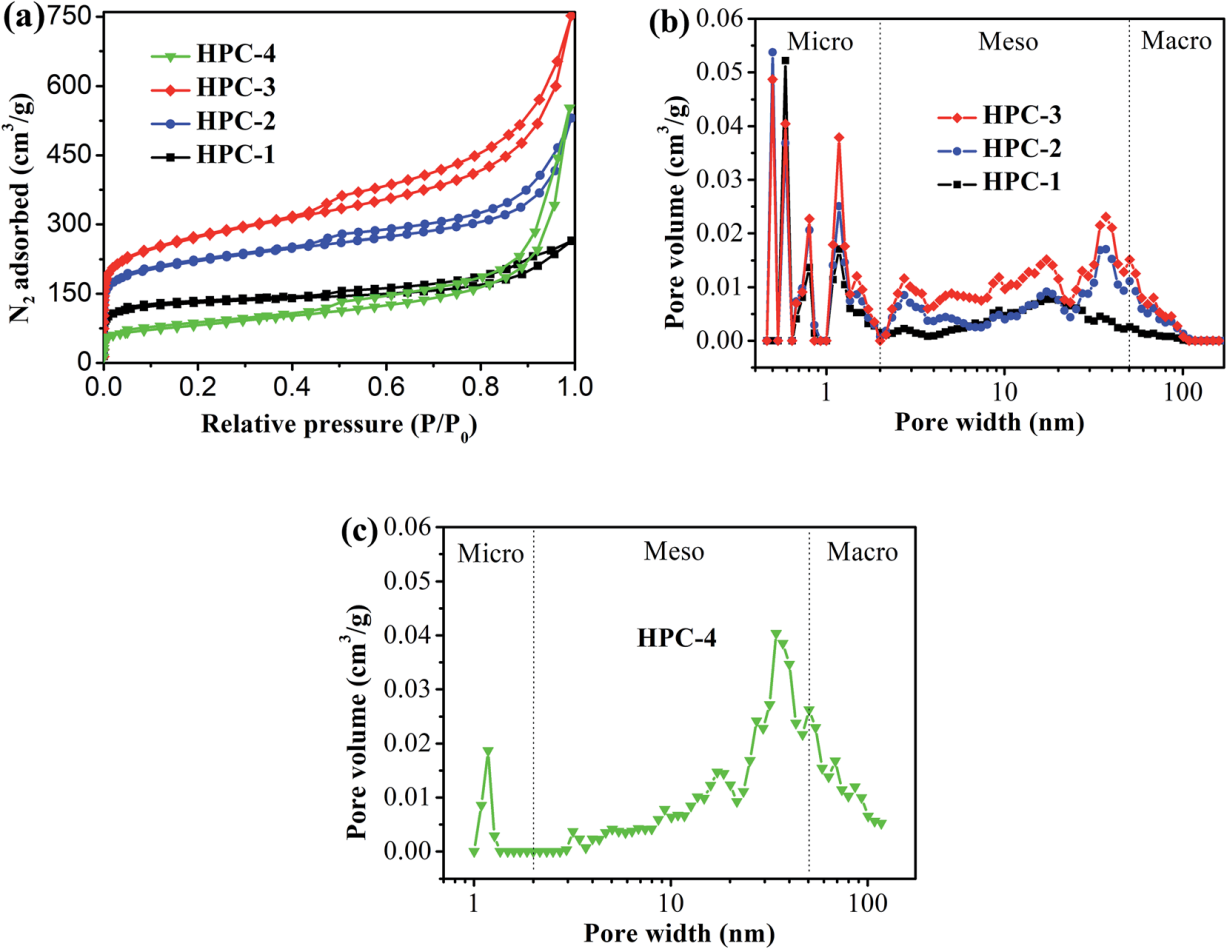
(a) Nitrogen adsorption/desorption isotherm of HPC samples, and pore-size distribution curves of (b) HPC-1, HPC-2 and HPC-3, and (c) HPC-4.

Fig. 2 shows the typical nitrogen (77 K) adsorption/desorption isotherm and pore-size distribution curves of the HPC samples. The isotherms for all samples exhibit typically type IV isotherms according to the IUPAC classification (see Fig. 2(a)).^43^ The sharp N_2_ uptake at low pressure (*P*/*P*_0_ < 0.1) for HPC samples indicates the existence of abundant micropores.^44^ The samples also show a type-H_1_ hysteresis hoop at the range of 0.45–1.0 and obvious capillary condensation steps, suggesting the existence of a pore size range from mesoporous to macropores.^41,45^ The pore size distribution plots were derived from the adsorption branch of the isotherm based on the density functional theory (DFT). The HPC samples contain abundant micropores, mesopores and macroporous (see Fig. 2(b and c)), indicating that the obtained HPC samples possessed a hierarchical pore structure.

The detailed pore parameters for the HPCs are listed in Table 1. The specific surface area was calculated using the Brunauer–Emmett–Teller (BET) method based on adsorption data in the partial pressure (*P*/*P*_0_) range of 0.08–0.30, and the total pore volume was determined from the amount of nitrogen adsorbed at *P*/*P*_0_ = 0.99. As the weight ratio of trilithium citrate tetrahydrate to sucrose increases, the BET surface area and the total pore volume of HPCs increase and reach to the maximum value of 991 m^2^ g^−1^ and 1.50 cm^3^ g^−1^, respectively, at 1 : 2.31. Because the mesopores and macropores in HPCs are created by removing Li_2_CO_3_ nanoparticles, the mesopore and macropore volume increases drastically with the increase of trilithium citrate tetrahydrate, and contributing more than 77.3% of the total pore volume for HPC-3 sample. The micropore volume is almost independent of the change of trilithium citrate tetrahydrate, further confirmed that the gases (such as CO_2_, H_2_O, CO) released from the pyrolysis of the sucrose is responsible for the formation of micropores.

**Table 1:** Porosity parameters of HPC samples

Sample	Sucrose : lithium citrate (wt%)	*S*_BET_ (m^2^ g^−1^)	Pore volume (cm^3^ g^−1^)		
			*V*_t_	*V*_mic_	*V*_mes&mac_
HPC-1	1 : 0.77	412	0.62	0.26	0.36
HPC-2	1 : 1.54	708	1.08	0.34	0.74
HPC-3	1 : 2.31	991	1.50	0.34	1.16
HPC-4	1 : 3.08	289	1.26	0.06	1.20

**Fig. 3:**
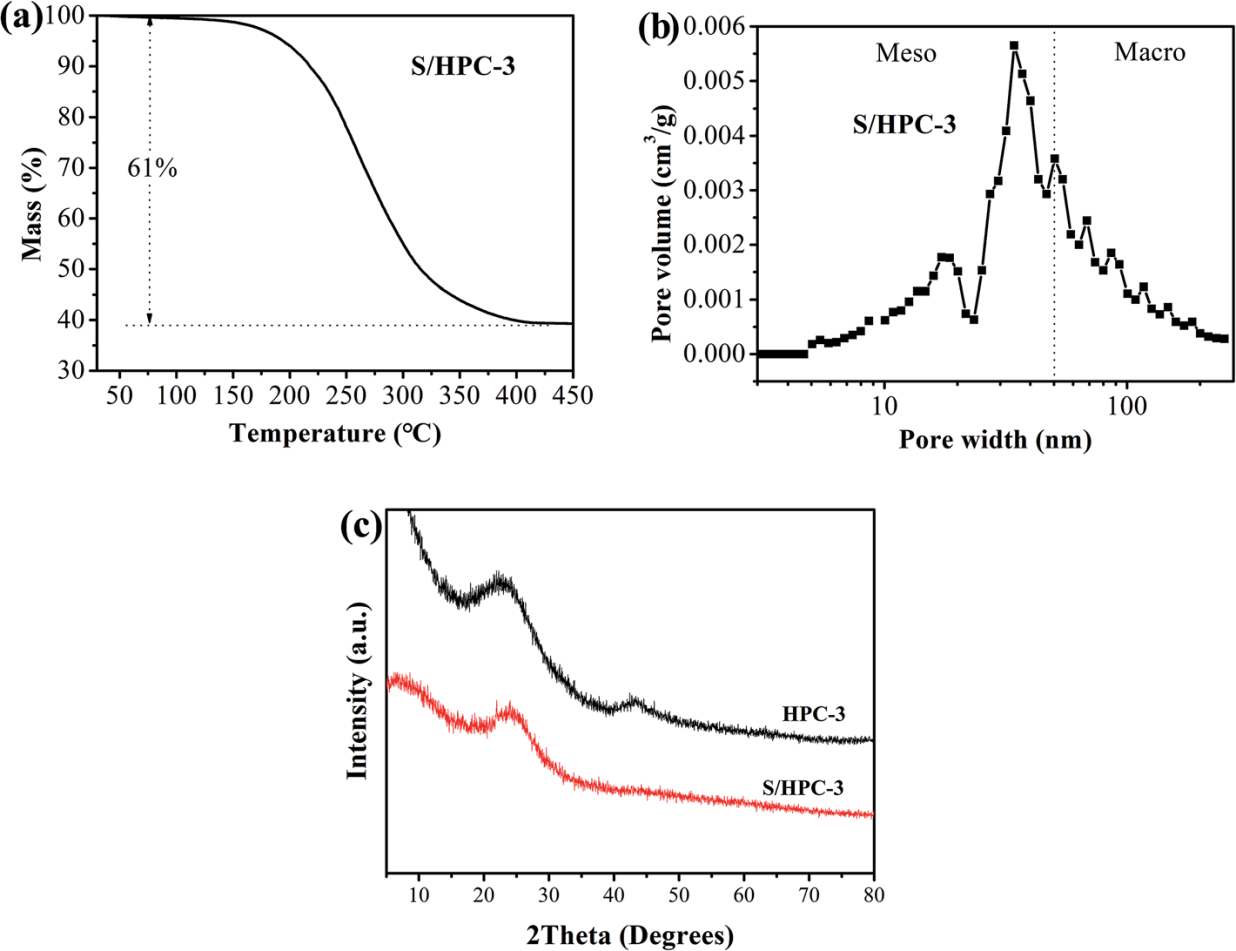
(a) Thermogravimetric curve of S/HPC-3 sample in N_2_, (b) pore-size distribution curve of S/HPC-3 composite, and (c) XRD patterns of HPC-3 and S/HPC-3 composite.

Due to the highest *S*_BET_ and the largest total pore volume, the as-prepared HPC-3 sample was chosen as the host to encapsulate sulfur for preparing cathode materials. Considering volume expansion, the theoretical amount of sulfur that HPC-3 can accommodate was calculated according to the following method: the loading amount of sulfur = weight of the HPC × total pore volume × density of lithium sulfide (1.66 g cm^−3^) × the weight ratio of sulfur in lithium sulfide (69.78%).^18^ Based on the total pore volume of HPC-3 (1.50 cm^3^ g^−1^), the theoretical amount of sulfur is calculated to be 63.3 wt%. Measured by the thermal gravimetric analysis (TGA), the sulfur content in the S/HPC-3 composite is actually about 61 wt%, as shown in Fig. 3(a), less than the theoretical limit of 63.3 wt%. The pore-size distribution curve of S/HPC-3 composite is displayed in Fig. 3(b). After encapsulating sulfur into hierarchical pores, the *S*_BET_ and total pore volume of S/HPC-3 composite decreased to 14 m^2^ g^−1^ and 0.16 cm^3^ g^−1^, respectively. The micropores are disappeared, further confirmed that the molten sulfur was accommodated into the pores of HPC-3 *via* capillary force.^46^ A portion of mesopores and macroporesis still available for S/HPC-3, which can help buffering volume expansion, allowing the accessibility of the electrolyte and fast transport of Li^+^ during cycling process. XRD patterns for HPC-3 and S/HPC-3 composite are illustrated in Fig. 3(c). The broad diffraction peaks around 23° and a weak peaks at 44° are observed in the pattern of HPC-3, indicating an amorphous state of carbon.^41^ The S/HPC-3 composite exhibits only one broad diffraction peak at 23°, and no diffraction peaks of the crystalline sulfur were observed, indicating that sulfur is confined in the internal pores.^41,47^

**Fig. 4:**
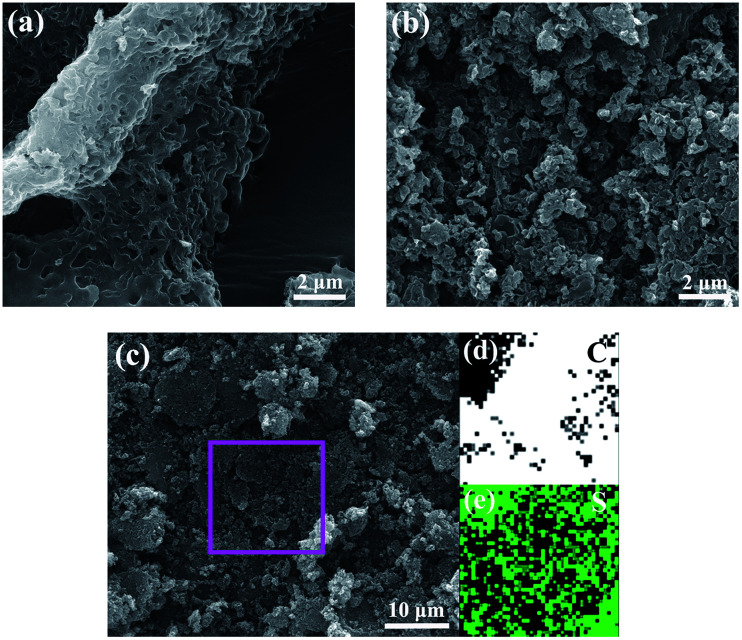
SEM image of (a) HPC-3 and (b) S/HPC-3, elemental mappings of (d) carbon and (e) sulfur corresponding to the area outlined by the magenta square in SEM image of (c) S/HPC-3.

Field-emission scanning electron microscopy (SEM) was used to examine the morphology of the HPC-3 and S/HPC-3 composite. As shown in Fig. 4(a), the HPC-3 samples exhibit highly developed porous structure, and the pores are three-dimensional and interconnected. Large mesopores or macropores are evenly distributed in carbon matrix, which is attributable to the *in situ* formation of Li_2_CO_3_ templates in carbon host during preparation. The special pore structure could favor for the sulfur encapsulation. After loading 61% sulfur, a portion of macropores are still observed from S/HPC-3 composite, as shown in Fig. 4(b), which can provide space for electrolyte ingress and fast Li^+^ transportation during cycling. Sulfur nanoparticles are locked in the inner pores of HPC-3, and no large bulk sulfur could be observed on the surface due to its high specific surface area and high pore volume. The elemental mapping images of carbon (Fig. 4(d)) and sulfur (Fig. 4(e)) corresponding to the area outlined by the magenta square in SEM image of S/HPC-3 (Fig. 4(c)) clearly demonstrate that sulfur has a highly dispersed state in HPC-3, which in accordance with the XRD results.

**Fig. 5:**
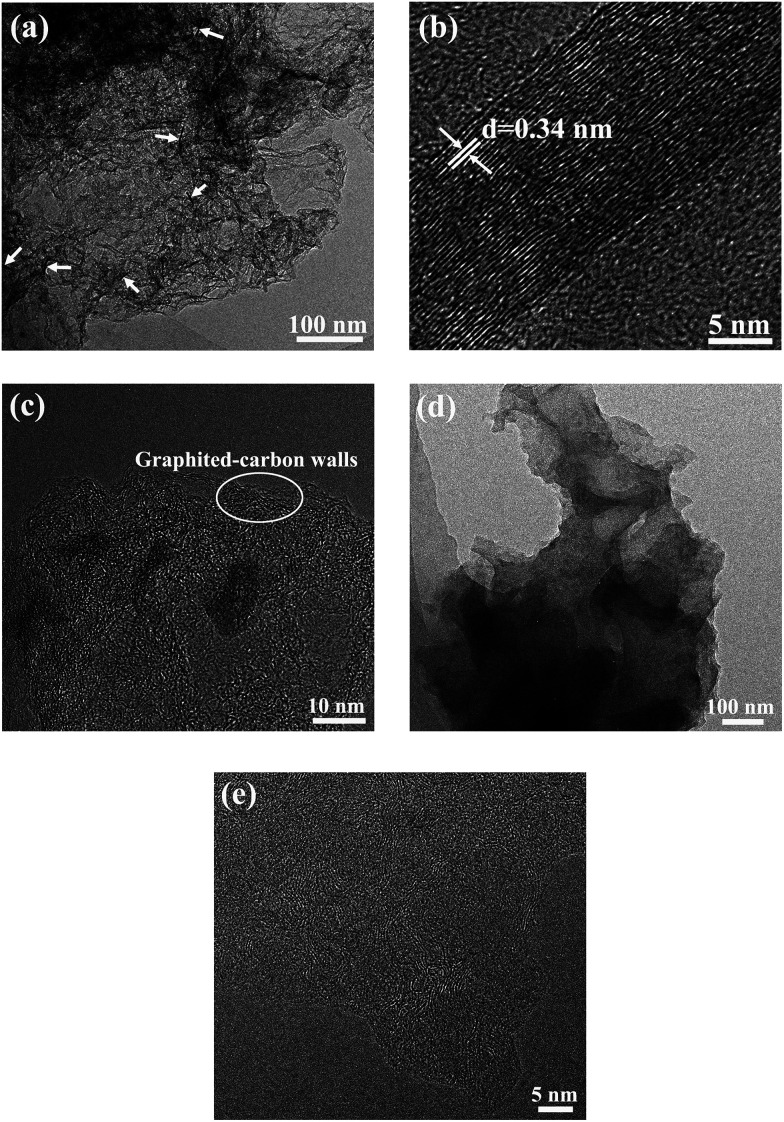
TEM images of (a) HPC-3 and (d) S/HPC-3, HRTEM images of (b) graphite structure, (c) HPC-3 and (e) S/HPC-3.

To further observe the morphology of the HPC-3 and S/HPC-3 composites, TEM measurements were introduced. As presented in Fig. 5(a), the HPC-3 sample consists of three-dimensionally interconnected pore structure and some laminated structures (marked with white arrows). The HRTEM image of the laminated structure (Fig. 5(b)) shows clear crystal plane with an interplanar distance of 0.34 nm, which is corresponds to the graphite (002) crystal plane. The similar graphite structures are also reported by Klose *et al.* who synthesized carbon materials by carbonizing iron-containing metal–organic framework.^48^ The HRTEM image of HPC-3 (Fig. 5(c)) further shows that most of these pores are irregular and the thicknesses of carbon walls are less than 4 nm. The carbon walls are consisted of localized graphitic structures and amorphous components. The space between two distorted lattice fringes is 0.34 nm, which is consistent with the (002) plane of partially graphitized HPC-3.^49^ These graphite flakes and localized graphited-carbon walls are both beneficial for electronic transmission. The carbon material and sulfur are both hydrophobic, and thus available for the ready absorption of sulfur into the pores through capillary forces. As displayed in Fig. 5(d), the sulfur nanoparticles (black dots) are evenly distributed in carbon host without any agglomerations of bulk sulfur on the surface, indicating the full incorporation of sulfur into the pores. This result is corresponding to the previous XRD, SEM, and nitrogen adsorption/desorption results, which strongly confirmed that the sulfur is completely encapsulated in the pores of HPC-3. As shown in HRTEM image of Fig. 5(e), the S/HPC-3 composite still remains the graphited-carbon walls. The highly dispersed sulfur encapsulated into the hierarchical pores of HPC-3 can generate intimate contact with interconnected carbon framework, giving rise to a short pathway for transport of both Li^+^ and electrons.

**Fig. 6:**
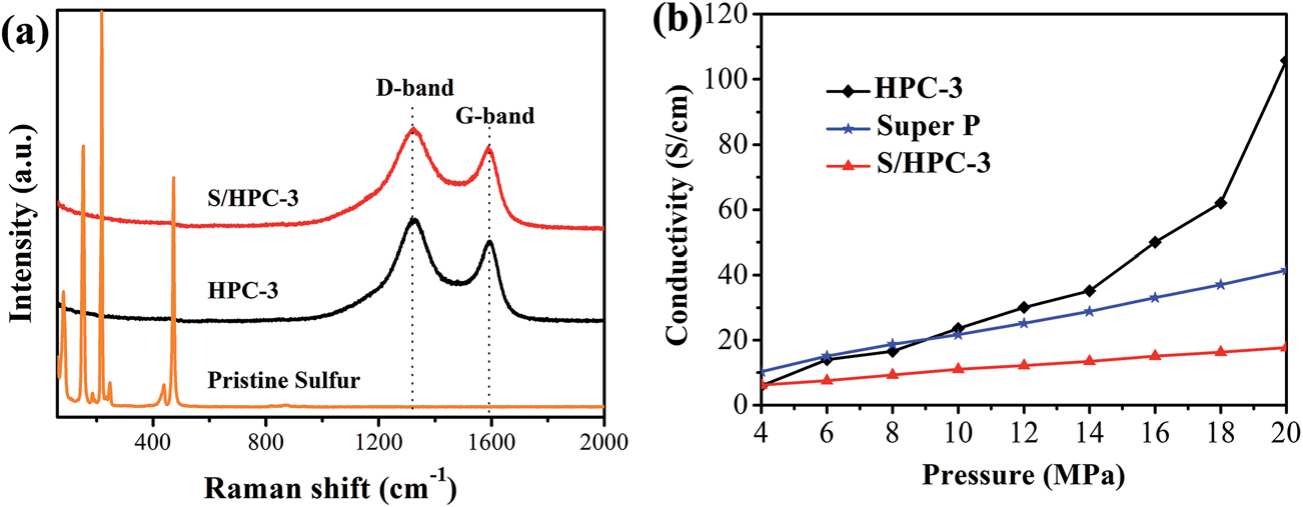
(a) Raman spectra of pristine sulfur, HPC-3 and S/HPC-3, and (b) electronic conductivity of HPC-3, S/HPC-3 composite and super P.

Raman spectroscopy is a very useful tool to study the microstructure of carbon in composite material. Fig. 6(a) compares the Raman spectrum of the pristine sulfur, HPC-3 and S/HPC-3 composites. For HPC-3 sample, two intense broad bands located at 1328 and 1587 cm^−1^ are attributed to the carbon defects (D-band) and the in-plane vibration of sp^2^ carbon atoms (G-band), respectively.^50^ The coexistence of two bands indicates that the carbon is partially graphitized with some defects and disorders. The calculated integral intensity ratios (*I*_D_/*I*_G_) is a useful index for comparing the degree of crystallinity of various carbon materials.^49^ In terms of the integral areas, we can calculate the *I*_D_/*I*_G_ ratio of the HPC-3 sample to be about 3.01. Compared with HPC-3, the *I*_D_/*I*_G_ ratio of S/HPC-3 composite is slightly increased to 3.37, suggesting more lattice defects, which arise from the merging of sulfur atoms into the HPC-3 lattice.^43^ Furthermore, Raman spectra of S/HPC-3 exhibits no characteristic peaks of elemental sulfur in the region of 60–500 cm^−1^, which are related to vibration of the S–S bond in S_8_ species.^51^ This phenomenon suggested that sulfur is successfully impregnated into pores and adsorbed on the internal surface of HPC-3 host, which is in good accordance with the XRD and N_2_ adsorption/desorption results.

To further confirm the microstructures and investigate the changes before and after sulfur encapsulating, the electronic conductivity of HPC samples and S/HPC-3 composite were tested by the four-probe method. The samples were loaded in a Teflon cylinder with an inner diameter of 16 mm, and two stainless steel plungers were used to deliver pressure through a hydraulic press device. The voltage and current through stainless-steel plungers were recorded by using two Keithley 2000 digital multimeters. The electrical conductivity was calculated on the basis of the powder electrical resistivity. Fig. 6(b) shows the conductivity values of HPC and S/HPC-3 samples at the pressure range of 4–20 MPa, respectively. In a control experiment, the electronic conductivity of Super P is also measured. The electronic conductivity is slightly higher for Super P when the applied pressure is less than 9 MPa. But with the pressure increase to 20 MPa, the electronic conductivity for HPC-3 is dramatically increased and reach to the maximum value of 105.7 S cm^−1^, which is more than two times higher than that of Super P (41.5 S cm^−1^). The high conductivity of HPC-3 at high pressure is related to the formation of graphitic and porous structure, which is confirmed by SEM, HRTEM and Raman spectroscopy. The electrical conductivity of the S/HPC-3 composite is lower than HPC-3, which is ascribed to the encapsulated sulfur. The comparison between the electrical conductivity of HPC samples and super P are also shown in Fig. S4.†

**Fig. 7:**
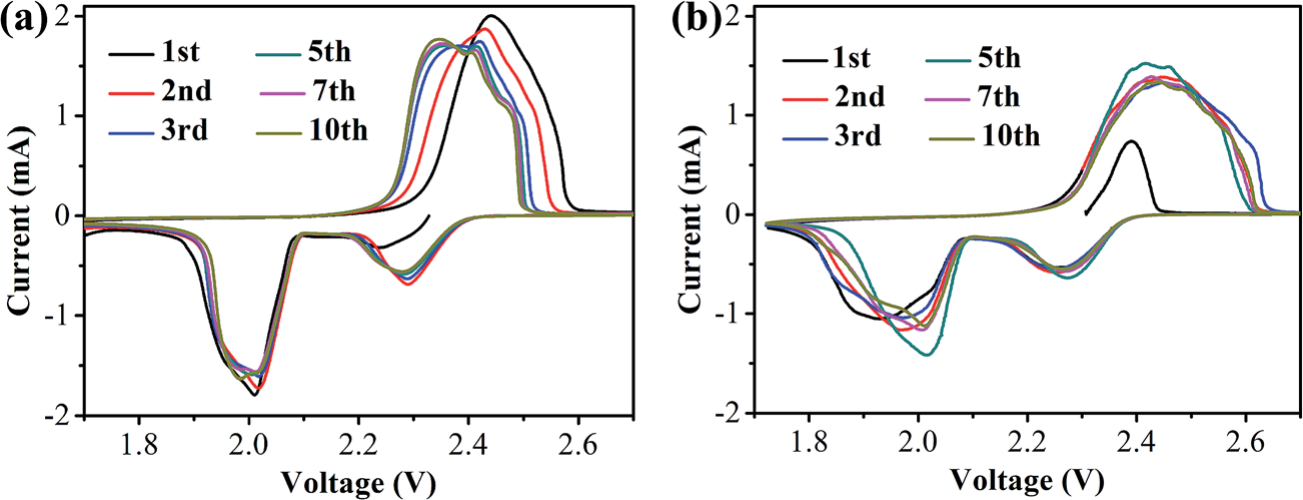
The CV curves of (a) S/HPC-3 and (b) pure sulfur cathode during the first 10 cycles.

CR2025 coin-type half-cells using lithium foils as the anode and the S/HPC-3 composite as the cathode were assembled to investigate the electrochemical properties. For comparison, the performance of the pure S cathode with 60 wt% sulfur content was also evaluated in the same conditions. Fig. 7(a) and (b) presents the cyclic voltammogram (CV) curves of the S/HPC-3 composite and pure S cathode in the voltage range of 1.7–2.7 V with a scan rate of 0.2 mV s^−1^, respectively. For S/HPC-3 composite cathode, two typical reduction (cathodic) peaks at approximately 2.29 V and 2.01 V were observed, corresponding to the reduction of sulfur to long-chain lithium polysulfides (Li_2_S_*n*_, 4 ≤ *n* ≤ 8) and further reduction to Li_2_S_2_ and eventually to Li_2_S, respectively.^52,53^ One oxidation peak (anodic) at about 2.44 V with shoulder at 2.47 V are attributed to the oxidization of solid Li_2_S and Li_2_S_2_ to lithium polysulfides and further conversion to element sulfur, respectively.^54,55^ After the first two cycles, the oxide peak splits into two peaks and gradually shift to lower potentials, and no distinct current changes for cathodic peaks, indicating a high degree of reactive reversibility and electrochemical stability of the S/HPC-3 cathode.^49^ Compared to pure S, the S/HPC-3 composite had a higher reduction peak current and oxidation peak current indicate that the S/HPC-3 composite had a higher charge/discharge capacity. In addition, the potential difference between the reduction and oxidation current peaks (Δ*E*) is an indicative of electrochemical polarization. The S/HPC-3 composite displayed a smaller Δ*E* than the pure S cathode, indicating a smaller electrochemical polarization. The large surface area and high pore volume of HPC-3 provide more reactive sites and unobstructed transport channels for Li^+^ and electrolyte, which can lower the electrochemical polarization and improves the utilization of sulfur (more capacity output).^53^

**Fig. 8:**
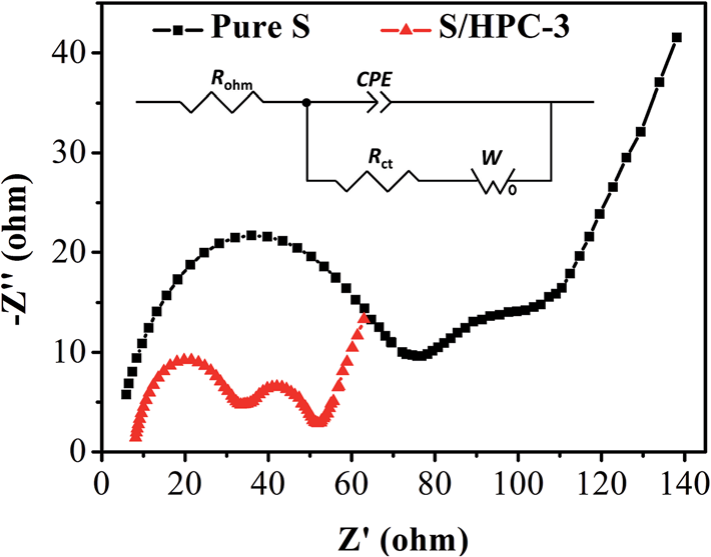
Electrochemical impedance spectrum of S/HPC-3 and pure sulfur in lithium/sulfur batteries before initial discharge.

Fig. 8 is the Nyquist plots of S/HPC-3 composite and pure S. The intercept at the real axis resistance is corresponding to the combined resistance (*R*_ohm_), which is determined by the ionic resistance of the electrolyte, the intrinsic resistance of the active materials, and the contact resistance at the active material/current interface.^15,36^ The depressed semicircle in the high-frequency region is corresponding to the charge-transfer resistance (*R*_ct_) through the electrode–electrolyte interface. The short inclined lines in the low frequency region are associated with a semi-infinite Warburg diffusion process of soluble lithium polysulfide in the electrolyte.^15,36^ It is obvious that the S/HPC-3 composite cathode shows a much smaller *R*_ct_ value (29 Ω) than pure S cathode (87 Ω), suggesting a lower charge-transfer resistance of composite cathode. Combining the results of CV, electric conductivity and BET, we speculate that the close contact between sulfur and the carbon host in the S/HPC-3 composite, and high electric conductivity of carbon host might be the reasons for the smaller *R*_ct_.

**Fig. 9:**
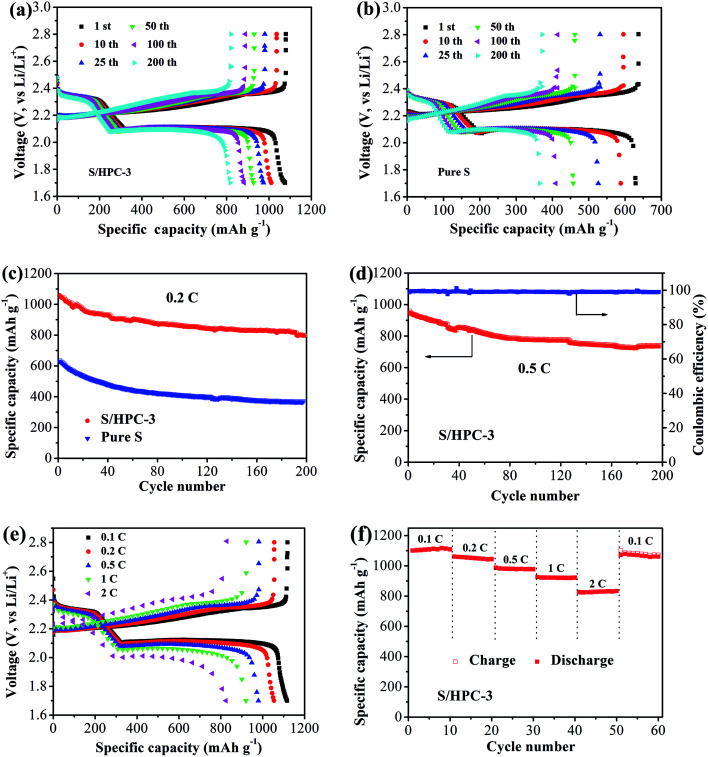
Galvanostatic charge–discharge curves of (a) S/HPC-3 and (b) pure S at 0.2C, (c) cycling performances of S/HPC-3 and pure S at 0.2C, (d) cycling performance of the S/HPC-3 at 0.5C, (e) galvanostatic charge–discharge curves of S/HPC-3 at different current density, and (f) rate performance of S/HPC-3 composite cathode.

Fig. 9(a) and (b) show the galvanostatic discharge/charge curves with S/HPC-3 composite and pure sulfur cathodes in the 1st, 10th, 25th, 50th, 100th and 200th cycles at 0.2C (1C = 1675 mA g^−1^) between 1.7 V and 2.8 V. All capacity values in this article are calculated on the basis of sulfur mass. Consistent with the CV results, the discharge voltage profiles of the two samples exhibit two typical discharge plateaus at around 2.34 V and 2.1 V, corresponding to the formation of high order polysulfide and Li_2_S_2_/Li_2_S, respectively. In order to reveal the overall electrochemical performance, the cyclic stability of S/HPC-3 composite and pure sulfur cathode was investigated, as shown in Fig. 9(c). The S/HPC-3 composite demonstrates a higher initial discharge capacity of 1059 mA h g^−1^ than that of pure sulfur cathode (640 mA h g^−1^) at a current density of 0.2C. Both composites show a rapid capacity loss in the beginning 30 cycles due to unavoidable dissolution of polysulfides into the electrolytes. In the following cycles, S/HPC-3 composite exhibits a relatively stable charge–discharge process. After 200 cycles, the S/HPC-3 composite still remains a capacity of 797 mA h g^−1^, much higher that of pure sulfur cathode (365 mA h g^−1^). The decay rate of S/HPC-3 and pure sulfur cathode is calculated to be 0.12% and 0.21% per cycle, respectively. To further investigate the high rate and long cycling life of the S/HPC-3 composite, a prolonged cycling test was conducted at a current density of 0.5C. As shown in Fig. 9(d), an initial discharge capacity of 975 mA h g^−1^ is delivered, and after 200 cycles, a discharge capacity of 735 mA h g^−1^ is still preserved on the S/HPC-3 composite cathode. The decay rate is 0.12% of initial capacity per cycle, thus exhibiting outstanding cycling stability and high capacity. The coulombic efficiency remains around 98.7% over the 200 cycles, thus indicating the very small impact of the shuttle effect of polysulfides. The higher discharge capacity and better cycling performance of S/HPC-3 could be attributed to its unique hierarchical porous structure available after sulfur loading and buffer volume expansion during cycling.

Fig. 9(e) exhibits the representative discharge–charge curves with S/HPC-3 composite cathode at various current rates. The discharge capacities at 0.1, 0.2, 0.5, 1, and 2C are 1119, 1056, 982, 921, and 829 mA h g^−1^, in their turns. It is noticeable that, with increasing current density, the space between the charge and discharge plateaus are enlarged. This phenomenon is due to the kinetic effects of the material, rendering a large polarization.^56^Fig. 9(f) displays the rate capabilities of the S/HPC-3 composite electrode from 0.1C to 2C. The discharge capacities maintain stable and reduce regularly with the increase of current rate. After every 10 cycles at a specific current rate, the capacities retention at 0.1, 0.2, 0.5, 1 and 2C are about 100.8%, 98.6%, 99.7% and 96.4% of the initial discharge capacities, implying an excellent rate cycling of the S/HPC-3 composite electrode. When the current density return to 0.1C, the discharge capacity can be recovered, which is 1060 mA h g^−1^. These results demonstrate that the S/HPC-3 composite electrode has good electrochemical reversibility and structural stability.

Based on the above results, the excellent electrochemical performance of S/HPC-3 composite cathodes can be attributed to the following two reasons: (1) the synergistic advantages of hierarchical porous structure. The macropores and large mesopores accommodate a high sulfur loading, provide unobstructed transport channels for Li^+^ and electrolyte, and accommodate the volume variation during charge/discharge process, leading to high discharge capacity and better cycling performance. The microporous and small mesopore can provide more active sites for electrochemical reaction, and trap sulfur and polysulfide, thereby alleviating the shuttle effect and improving the efficiency and stability. Additionally, three-dimensionally interconnected pore structures shorten the transport distance of Li^+^ and electrolyte, which is favorable for high rate capability. (2) High electronic conductivity. The localized graphited-carbon walls and graphite structures offers high electrical conductivity for composite cathode, which is also contribute to the excellent rate capability.

## 4. Conclusions

In this paper, HPC samples with micropores, mesopores and macrospores were synthesized by *in situ* formation of template in carbon matrix. HPC-3 sample with the highest specific surface area and the largest pore volume can be obtained by adjusting the ratio of trilithium citrate tetrahydrate to sucrose being 2.31 : 1. The HPC-3 sample consists of three-dimensionally interconnected pore structure with graphited-carbon walls and graphite flakes, which increase the electric conductivity of HPC-3. When encapsulating 61% sulfur into the HPC-3 host, the obtained S/HPC-3 composite cathode material displayed a high initial capacity of 1059 mA h g^−1^ and a stable capacity of 797 mA h g^−1^ after 200 cycles, at a current density of 0.2C. Moreover, the S/HPC-3 composite cathode delivered a high specific capacity of 975 mA h g^−1^ and a stable capacity of 735 mA h g^−1^ after 200 cycles, at a current density of 0.5C. The discharge capacities of S/HPC-3 composite cathode at 0.1, 0.2, 0.5, 1, and 2C are 1119, 1056, 982, 921, and 829 mA h g^−1^, in their turns. The excellent electrochemical performance of S/HPC-3 is attributed to the synergistic advantages of hierarchical porous structure and high electronic conductivity.

## Conflicts of interest

There are no conflicts to declare.

## Supplementary Material

RA-008-C7RA12978E-s001
