# Supplementary material for: In situ template synthesis of hierarchical porous carbon used for high performance lithium–sulfur batteries
Source: RSC Adv. 2018 Jan 24;8(9):4503–13. doi: 10.1039/c7ra12978e (PMC9077842; doi:10.1039/c7ra12978e)
Supplement: RA-008-C7RA12978E-s001 [file RA-008-C7RA12978E-s001.pdf]

Electronic Supplementary Material (ESI) for RSC Advances.
This journal is © The Royal Society of Chemistry 2018

In-situ Template Synthesis of Hierarchical Porous Carbon Used for High Performance Lithium-sulfur Batteries

Lizhen Long[a,*], Xunyuan Jiang[b], Jun Liu[a], Dongmei Han[b,*], Min Xiao[b], Shuanjin Wang[b], Yuezhong Meng [b,*]

[a] *College of Physics and Technology, Guangxi Normal University, Guilin 541004, PR China*
[b] *The Key Laboratory of Low-carbon Chemistry & Energy Conservation of Guangdong Province/State Key Laboratory of Optoelectronic Materials and Technologies, Sun Yat-sen University, Guangzhou 510275, PR China*

*Corresponding author: Telephone & Fax: +86-20-84114113
E-mail: mengyzh@mail.sysu.edu.cn (Yuezhong Meng)
handongm@ mail.sysu.edu.cn
longlzh@foxmail.com

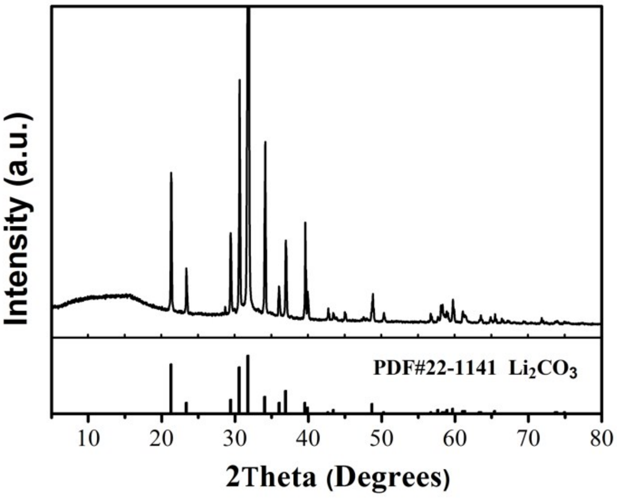

Figure S1. XRD pattern of HPC-3 before acid-treatment.

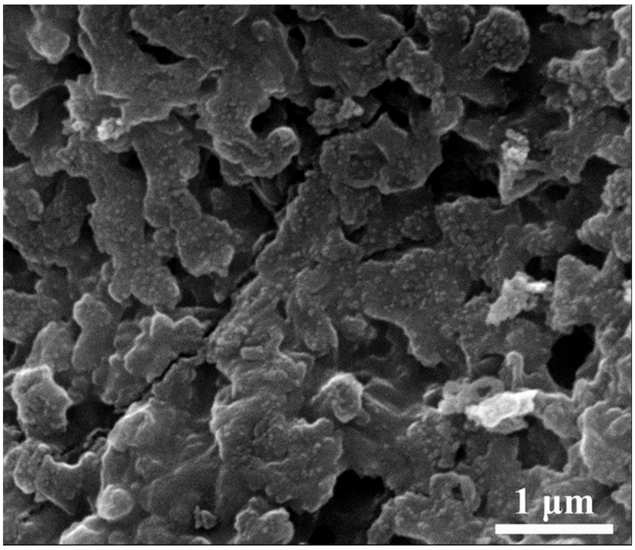

Figure S2.SEM image of HPC-3 before acid-treatment.

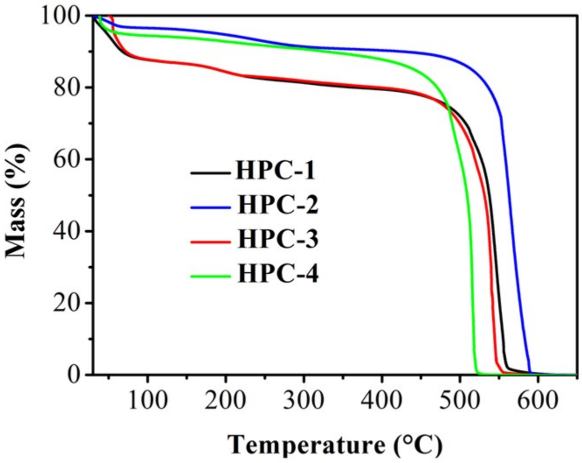

Figure S3 Thermogravimetric analysis of HPC-1, HPC-2, HPC-3 and HPC-4 in air.

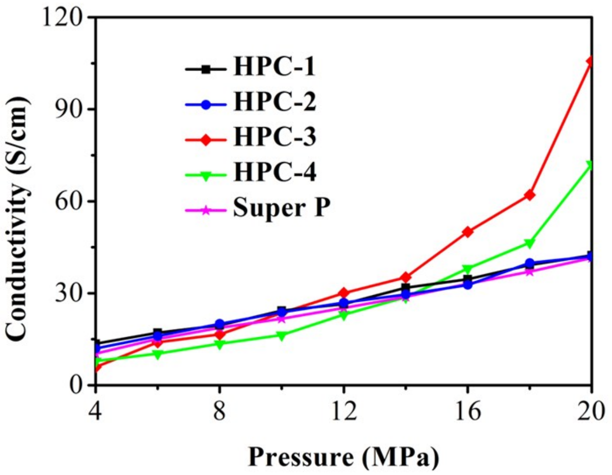

Figure S4. Electric conductivity of HPCs samples and super P.
